# The Value of Preventative Dental Care: A Discrete-Choice Experiment

**DOI:** 10.1177/0022034521989943

**Published:** 2021-02-04

**Authors:** D. Boyers, M. van der Pol, V. Watson, T. Lamont, B. Goulao, C. Ramsay, A. Duncan, L. Macpherson, J. Clarkson

**Affiliations:** 1 Health Economics Research Unit, University of Aberdeen, Aberdeen, UK; 2 School of Dentistry, University of Dundee, Dundee, UK; 3 Health Services Research Unit, University of Aberdeen, Aberdeen, UK

**Keywords:** oral hygiene advice, scale and polish, prevention, economics, willingness to pay, stated preference

## Abstract

Scale and polish (SP) and oral hygiene advice (OHA) are commonly provided in primary care dental practice to help prevent periodontal disease. These services are widely consumed by service users, incurring substantial cost, without any clear evidence of clinical benefit. This article aims to elicit general population preferences and willingness to pay (WTP) for preventative dental care services and outcomes. An online discrete-choice experiment (DCE) was completed by a nationally representative sample of the UK general population. Respondents each answered 10 choice tasks that varied in terms of service attributes (SP, OHA, and provider of care), outcomes (bleeding gums and aesthetics), and cost. Choice tasks were selected using a pivoted segmented experimental design to improve task realism. An error components panel logit model was used to analyze the data. Marginal WTP (mWTP) for each attribute and level was calculated. In total, 667 respondents completed the DCE. Respondents valued more frequent SP, care provided by a dentist, and personalized OHA. Respondents were willing to pay for dental packages that generated less frequent (“never” or “hardly ever”) bleeding on brushing and teeth that look and feel at least “moderately clean.” Respondents were willing to pay more (+£145/y) for improvements in an aesthetic outcome from “very unclean” (−£85/y) to “very clean” (+£60/y) than they were for reduced bleeding frequency (+£100/y) from “very often” (−£54/y) to “never” (+£36/y). The general population value routinely provided SP, even in the absence of reductions in bleeding on brushing. Dental care service providers must consider service user preferences, including preferences for both health and nonhealth outcomes, as a key factor in any service redesign. Furthermore, the results provide mWTP estimates that can be used in cost-benefit analysis of these dental care services.

## Introduction

Health care systems aim to provide person-centered care. Health care funders therefore require information about the aspects, or attributes, of care that service users value and the trade-offs they are willing to make between different attributes of their care. For example, service users may prefer to receive a scale and polish (SP) from a dentist rather than a hygienist, but they may be willing to trade off care provider for reduced cost. Service users may value aesthetic outcomes and may even be willing to trade this against clinical benefits.

In 2018–2019, National Health Service (NHS) Scotland provided over 2 million simple SP treatments at a cost of £31 million (ISD Scotland 2019). However, recent studies show no evidence of clinical benefit of widespread provision of SP or of personalized oral hygiene advice (OHA) for adults who routinely attend primary dental practice and do not already show signs of severe periodontal disease (Lamont et al. 2018). It remains unknown how the general population values these services and what service attributes are most important.

People might value outcomes of care that are not well captured by clinical outcome measures, such as bleeding on probing. Clinical outcomes alone cannot account for the value associated with continuity of care, feelings of empowerment, or aesthetic and social benefits of having teeth that look and feel clean and healthy. It is important to gain a more holistic understanding of how the general population values dental services, including both clinical and nonhealth outcomes such as aesthetics, to inform patient-centered service configuration. The inclusion of nonhealth outcomes in economic valuation studies is increasingly recognized as important for decision making. Recent guidance suggests a reference case approach that encapsulates the quantification of patient relevant health and nonhealth effects of interventions to inform decision making (Sanders et al. 2016). Discrete-choice experiments (DCEs) are a commonly used survey-based economic method to elicit preferences that can incorporate all sources of value (health and nonhealth) to service users. The method is well established in valuing health and care (Clark et al. 2014; Soekhai et al. 2019), but its application within oral health remains rare (Barber et al. 2018).

The aim of this study is to use a DCE to elicit UK general population preferences for SP and personalized OHA services and relevant dental health and aesthetic outcomes.

## Methods

DCEs are grounded in economic theory, which assumes that the value of a good or service depends on the value of its different component attributes (Lancaster 1966). In DCEs, respondents make a series of choices between 2 or more hypothetical service configurations that vary in terms of their attributes (e.g., service delivery, health and nonhealth outcomes) in a manner that maximizes their level of satisfaction or utility contingent on the attribute’s levels. Trade-off information can help guide service redesign to ensure that services are in line with preferences. If the cost of the service is included as an attribute, then a monetary valuation, willingness to pay (WTP), for any configuration of a service’s attributes can be calculated.

The DCE described here aimed to elicit general population (potential service user) preferences for 2 dental care services: OHA and SP. The DCE was carried out in parallel with the Improving the Quality of Dentistry (IQuaD) randomized controlled trial (Ramsay et al. 2018) and used to value trial outcomes. The trial aimed to determine the clinical effectiveness and cost-benefit of offering different SP frequencies (none, 6-monthly, 12-monthly) alone or in combination with detailed personalized OHA to improve periodontal health of dentate adults in general dental practice. Further details of the DCE design process can be found in the Appendix.

### Selection of Attributes and Levels

The attributes and levels (Table 1) were identified and refined following best practice methods, using literature reviews, focus groups (Coast et al. 2012), and engagement with practicing dentists, hygienists, and service users to ensure that the DCE attributes and levels were clinically relevant, useful to policy makers, and meaningful to service users. Further detailed information on the literature review and focus groups has been provided as an appendix. The qualitative work played a crucial role in the final selection of attributes and levels. Provider of care (dentist/hygienist) was added to the SP frequency and provision of OHA attributes as the dental health professional providing the service was important in both the literature review and focus groups. Bleeding was framed as “bleeding when brushing your teeth” because focus group participants felt this was more relevant to them than the clinical “bleeding on probing” assessment at the dental practice. Aesthetic outcome was included as an attribute because focus group participants deemed this an important reason to have a SP. The DCE included a cost attribute, payable annually as an out-of-pocket payment over 3 y, to enable calculation of WTP. The levels of the cost attribute were derived from several sources: 1) from a payment card administered to the focus group participants (see Appendix), 2) from a similar payment card exercise included with a separate group of participants within a clinical trial, and 3) based on the maximum value of an SP in the private market, assuming 2 SPs delivered annually at a maximum cost of £75 per SP. The upper level of the cost attribute of £200 per year was chosen as the maximum value to comfortably cover the private cost of these services and to allow additional valuation for health and aesthetic outcomes. Full descriptions of the attributes and levels are provided in the Appendix.

**Table 1.** Final List of Attributes and Levels Included in the Discrete-Choice Experiment.

Attribute	Levels
OHA	No detailed and personalized advice
	Detailed and personalized advice (dentist)
	Detailed and personalized advice (hygienist)
SP	None
	1 per year (dentist)
	1 per year (hygienist)
	2 per year (dentist)
	2 per year (hygienist)
Bleeding	Never
	Hardly ever
	Occasionally
	Fairly often
	Very often
Aesthetics	Very unclean
	Unclean
	Moderately clean
	Clean
	Very clean
Cost	£10 per year
	£20 per year
	£50 per year
	£100 per year
	£200 per year

OHA, oral hygiene advice; SP, scale and polish.

### Designing the Choice Tasks

There are 1,875 different potential combinations (3^1^ × 5^4^) of attributes and levels, leading to over 1.76 million unique choice sets. A D-optimal experimental design with vague priors was created using NGENE experimental design software to reduce the number of DCE questions to 30 (ChoiceMetrics 2014). The design code is provided in the Appendix. The 30 choice tasks were further split into 3 blocks of 10. Each respondent completed 1 block of 10 choice tasks to minimize respondent burden. Each choice task included 2 different dental care packages and an opt-out (no dental care package) alternative. The “no dental care” package and the levels of 2 attributes (bleeding and aesthetics) were respondent specific to ensure that the tasks were realistic to respondents’ current dental health. This was achieved using a pivoted and segmented design (Rose et al. 2008). The levels of the outcome attributes in the dental care packages were pivoted around the respondent-specific opt-out reference level, meaning that respondents had an equal probability of being presented with dental care packages with improved, same, and worse outcome levels compared to the opt-out. An additional repeated choice task was added to check the consistency of responses. However, following best practice recommendations from the literature, respondents who failed the consistency test were not excluded from the estimation sample (Lancsar and Louviere 2006; Ryan et al. 2009). The Figure illustrates an example choice task.

**Figure.**
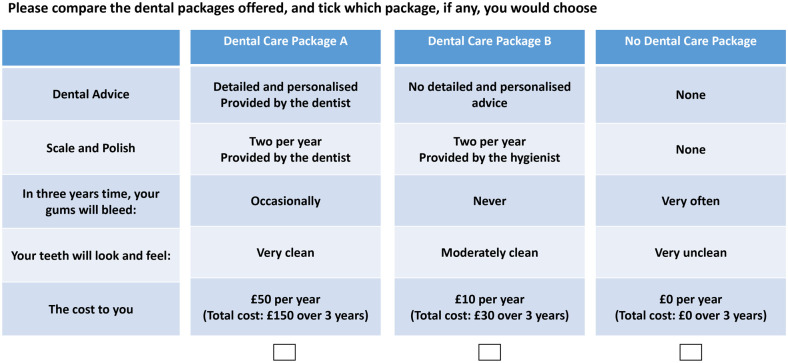
Example choice task from the discrete-choice experiment.

### Questionnaire Development

The survey comprised 3 sections. Section 1 asked respondents questions about their use, experience, and knowledge of dental care services. Section 2 described the choice tasks, explained the attributes and levels, described the implications of choosing the opt-out alternative, provided a completed example, and asked respondents to complete 10 choice tasks. Detailed instructions on how to complete the choice tasks, including elaboration on what is and is not included in a dental care package, are provided in the Appendix. Section 3 included demographic questions (age, sex, smoking status, UK region, income, education, and work status) to contextualize the results. The questionnaire was designed using an online survey development platform (Qualtrics), tested using a think-aloud study (further details provided in Appendix), and soft-launched with *N* = 30 respondents.

### Data Collection: Sample and Setting

The DCE was a self-administered online survey of a nationally representative sample of the UK general population. The sample size (*N* = 600) for the DCE required a minimum of *N* = 30 to 100 for each predetermined subgroup. Prespecified, planned subgroup analyses were conducted for participant characteristics by sex (male/female), UK region (Scotland/rest of UK), income (high/low), smoking status (smoker/nonsmoker), experience of SP (yes/no), and familiarity with dental hygienists (yes/no). Fifty respondents were sought across each of the 12 subgroups, allowing individuals to be present in more than 1 group. Population census-based quotas were used for age (among adult population), sex, and UK region. We oversampled in Scotland (*N* = 125) to enable subgroup analysis across regional specific payment systems. Data collection was conducted through Qualtrics, a web-based survey development platform and managed access panel provider (www.qualtrics.com). Survey respondents were recruited through a partner provider, who reimbursed respondents for their participation. The College Ethics Review Board at the University of Aberdeen, UK granted ethical approval (REF: 2015/12/1278).

### Data Analysis

Following random utility theory (McFadden 1973), each respondent (*n*) chooses their preferred (utility maximizing) dental care package (*j*) across the 3 alternatives in each of the 10 choice tasks (*t*). The data were analyzed using an error components panel logit model to estimate the relative importance of the attributes and levels (McFadden and Train 2000). Survey probability weights for country were used to account for overrepresentation of the sample in Scotland.

The observable component of the utility of the dental care packages (*V_njt_*) is specified as a linear and additive function of the attributes and levels presented to the respondents, where

(1) $$\begin{array}{l} V_{njt}=\alpha +\beta_{1}Advice_{dentist}+\beta_{2}Advice_{hygienist}\\ +\beta_{3}SP_{one\;from\;dentist}+\beta_{4}SP_{one\;from\;hygienist}\\ +\beta_{5}SP_{two\;from\;dentist}+\beta_{6}SP_{two\;from\;hygienist}\\ +\beta_{7}Bleed_{hardly\;ever}+\beta_{8}Bleed_{occasionally}\\ +\beta_{9}Bleed_{fairly\;often}+\beta_{10}Bleed_{very\;often}\\ +\beta_{11}Look_{unclean}+\beta_{12}Look_{moderately\;clean}\\ +\beta_{13}Look_{clean}+\beta_{14}Look_{very\;clean}\\ +\beta_{15}Cost_{annual} \end{array}$$

The alternative specific constant (α) is a random, normally distributed parameter, reporting mean and standard deviation reflecting the observed utility obtained from opting to choose a package of dental care as opposed to no package of care. All categorical variables β_1_ to β_14_ were included using effects coding, where the coefficient, β, represented the mean effect of an attribute level on the utility derived from the dental care package. The interpretation of β depends on the unit of measurement of the attribute as described in Table 1. For example, β_5_ represents the effect on utility of having 2 SPs per year from a dentist, relative to the grand mean of all the levels across that attribute. Positive coefficients indicate that packages containing this attribute level lead to a gain in utility, whereas negative coefficients indicate a loss in utility (relative to the grand mean). β_15_ indicates the linear impact of a £1 annual increase in the cost of the dental care package on overall utility.

Marginal WTP (mWTP) for a change in the level of each attribute (*k*) was calculated as the marginal rate of substitution between the attribute level coefficient and cost, such that $mWTP_{k}=-\beta_{k}/\beta_{cost}.$ For example, −β_5_/β_15_ indicates the annual WTP to have a dental care package consisting of 2 SPs from the dentist, all else held equal. Confidence intervals around mWTP estimates were calculated using the delta method. Further details of the subgroup analysis are provided in the Appendix. All analyses were performed using Stata V.14 (StataCorp) software.

## Results

In total, 667 respondents completed all 10 choice tasks, leading to 20,010 observations in the data set (667 participants × 10 completed choice tasks × 3 choice task alternatives), and 535 (80%) respondents passed the in-built consistency test, indicating that responses to the DCE had a high level of internal consistency. Dental care package A, package B, and the opt-out alternative were chosen in 37.7%, 39.5%, and 22.8% of choice tasks, respectively, indicating that respondents were willing to make trade-offs across the choice task alternatives. Only 1 respondent (<0.1% of the sample) always chose the opt-out, and no respondents always chose the same opt-in package. This provides reassurance that respondents traded between all the alternatives in the choice tasks. Median (interquartile range) survey completion time was 17 (13 to 24) min. Sample characteristics are outlined in Table 2.

**Table 2.** Sample Characteristics and Representativeness of the UK General Population.

Characteristic	Sample		General Population^a^
	No. (*N* = 667)	%	%
Age, y, mean ± SD	51 (16)		47
Sex			
Male	308	46	49
Female	359	54	51
Residence			
England	482	72	84
Scotland	125	19	8
Wales	44	7	5
Northern Ireland	14	2	3
Isle of Man	2	<1	<1
** **Currently a smoker			
Yes	98	15	18
No	447	67	59
Previously a smoker	122	18	23
Annual gross income^b^			
<£20,800	247	37	54
£20,800 to £41,600	211	32	37
≥£41,600	119	18	8
Prefer not to answer/blank	90	13	NA
Educational attainment			
O levels/SVQ (level 1 or 2)/1 A level	183	27	29
2+ A levels/SVQ (level 3)	101	15	12
Degree	164	25	27
Professional qualifications	81	12	6
Apprentice qualification	13	2	4
Vocational/foreign/other/none	125	19	22
Employment			
Any paid employment	326	49	61
Unemployed or seeking work	24	4	4
Retired	185	28	14
Student	20	3	9
Other	112	17	11
Self-reported dental health			
Very poor	6	1	1
Poor	28	4	6
Fair	200	30	21
Good	315	47	47
Very good	118	18	24
Self-reported general health			
Very poor	10	2	1
Poor	47	7	4
Fair	169	25	13
Good	343	51	34
Very good	98	15	47
Registered with a dental practice			
Yes	636	95	91
No	26	4	8
Don’t know	5	1	1
Normally pay for dental care			
Cocharge	307	46	45
NHS pays all cost	127	19	23
Private^c^	220	33	27
Never had dental care	6	1	2
Don’t know	7	1	1
Ever been to visit a dental hygienist			
Yes	414	62	47
No	213	32	53
Don’t know	40	6	<1
Normally have a SP every			
>3 mo	10	2	1
3 mo	65	10	6
6 mo	309	47	41
12 mo	121	18	19
24 mo	22	3	7
<24 mo	46	7	7
Never	84	13	18
Regular attendance^d^			
At least every 24 mo	570	86	NR
Less often	94	14	

NA, not applicable; NHS, National Health Service; NR, not reported; SP, scale and polish; SVQ, Scottish Vocational Qualifications.

a General population assumed of adults aged 16+ y in the United Kingdom calculated as 53,240,572 (Office for National Statistics [ONS] 2016).

b Excluding nonrespondents, 43%, 37%, and 21% of the sample were in the <£20,800, £20,800 to £41,600, and ≥£41,600 income brackets, respectively.

c Private dental care includes respondents reporting any private treatment, including out of pocket, insurance, and dental plan payments.

d Three respondents had missing data on this question; population-level data not reported.

Most respondents were regular dental attenders, had experience of SP (usually biannual or annual), and had experienced at least some treatment from a hygienist. Most never smoked and were in fair or better general and dental health. The sample closely matched the intended general population demographics on age and gender, with successful overrecruitment in Scotland as intended. The recruited sample had a lower proportion of smokers, had a higher proportion of people reporting good general health, and were from higher income categories compared to the general population. They were more likely to be regular dental attenders with experience of SP and receiving care from hygienists. Table 3 provides the results of analysis and mWTP estimates for changes in the attribute levels.

**Table 3.** Model Results and WTP for Dental Care Services and Outcomes.

Attribute/Level	β	Marginal WTP^a^	
		Mean	(95% CI)
Personalized advice from			
None^b^	−0.135***	−£12.84	(−£18.19, −£7.49)
Dentist	0.116***	£11.05	(£5.97, £16.14)
Hygienist	0.019	£1.79	(−£3.02, £6.60)
SP			
None^b^	−0.718***	−£68.20	(−£78.67, −£57.73)
12 mo (dentist)	0.182***	£17.25	(£9.03, £25.47)
12 mo (hygienist)	−0.062	−£5.87	(−£13.29, £1.56)
6 mo (dentist)	0.317***	£30.05	(£21.53, £38.58)
6 mo (hygienist)	0.282***	£26.77	(£19.31, £34.23)
Bleeding gums			
Never	0.392***	£37.25	(£26.22, £48.27)
Hardly ever	0.299***	£28.41	(£18.18, £38.63)
Occasionally	−0.032	−£3.08	(−£11.11, £4.95)
Fairly often	−0.090	−£8.54	(−£20.92, £3.84)
Very often^b^	−0.569***	−£54.04	(−£76.79, −£31.29)
Teeth look and feel			
Very unclean	−0.907***	−£86.09	(−£115.22, −£56.97)
Unclean	−0.413***	−£39.25	(−£52.34, −£26.16)
Moderately clean	0.147***	£13.98	(£3.98, £23.97)
Clean	0.532***	£50.56	(£38.35, £62.77)
Very clean^b^	0.641***	£60.82	(£47.6, £74.03)
Cost			
Annual	−0.011***		
Alternative specific constant (ASC)			
Mean	0.471***	£44.72	(£28.44, £61.00)
SD	1.480***		
Log-likelihood	−4,617.42		
Observations (*N*)	20,010		
Respondents (*N*)	667		
AIC	9,268.84		
BIC	9,403.21		

AIC, Akaike information criterion; BIC, Bayesian information criterion; WTP, willingness to pay.

a WTP estimates should be interpreted as the general population’s mean value of each attribute and level. The differences between WTP values indicate how much the general population values moving from one dental care package to another. For example, the general population would be willing to pay an additional £91.29 per year, over 3 y, to move from having bleeding gums very often (very often: −£54.04) to never having bleeding gums (never: +£37.25). They would be willing to pay £145.50 per year to move from having teeth that look and feel very unclean to teeth that look and feel very clean.

b Reference level used in the effects coded model.

*** *P* < 0.01.

The alternative specific constant (ASC) has a positive coefficient, indicating that the general population prefers to have a dental care package compared to none. Statistically significant positive coefficients show that the general population prefers dental care packages that generate less frequent bleeding and aesthetic improvements. They also prefer 6-monthly SP (all providers), 12-monthly SP (dentist only), and personalized OHA (dentist only), regardless of the bleeding or aesthetic outcomes as well as packages that cost less money. The general population is willing to pay more for care provided by a dentist than a hygienist, more for SP than personalized OHA, and more for improvements in aesthetic than bleeding outcomes.

Table 4 shows an example of how the DCE output can be used to identify important trade-offs in service design. The total WTP (sum of mWTP across all attributes) for package A is £47.81 while the WTP for package B is £38.80. Package A is preferable in terms of the interventions delivered, but package B has better outcomes overall. When considering all of these trade-offs, package A is preferred in this instance because the value derived from increased services outweighs the value derived from the better outcomes in Package B.

**Table 4.** WTP for Dental Packages.

Package A		Package B	
Package	+£44.72	Package	+£44.72
6 mo SP (hygienist)	+£26.77	No SP	−£68.20
No personalized advice	−£12.84	Personalized advice from dentist	+£11.05
Bleeding gums—hardly ever	+£28.41	Bleeding gums—never	+£37.25
Teeth look and feel—unclean	−£39.25	Teeth look and feel—moderately clean	+£13.98
Total WTP per year	£47.81		£38.80

SP, scale and polish; WTP, willingness to pay.

## Discussion

This study is the first DCE to investigate general population preferences for primary dental care services. The results show that the general population value both SP and personalized OHA even in a model that controls for frequency of bleeding gums on brushing and aesthetics (look and feel of teeth). Our results provide several important findings for policy makers regarding the organization of services. The general population values SP more highly than personalized OHA. If a choice needs be made about what service to provide (and they are equally effective in terms of bleeding gums and aesthetics), then the general population (the set of all potential service users) prefers SP over personalized OHA. They also prefer some of these services (personalized OHA and 12-monthly SP) to be delivered by the dentist rather than hygienist. This is important to take into account when allocating care responsibilities within the dental team. Care provided by the dentist is generally more costly to provide than care provided by a hygienist. Our estimates of the differences in WTP can help inform whether or not more costly care is justified by the additional value that is placed on that care.

A unique strength of this study is that the mWTP estimates can be used to inform cost-benefit analyses (CBAs) of any potential configuration of dental care service that comprises the attributes included in the DCE. CBA allows decision makers to consider a wider range of evidence, beyond narrowly defined, short-term health outcomes such as bleeding gums that are insensitive to the processes and outcomes of care that are valued by dental service users (Kastenbom et al. 2019). The approach allows integration of patients’ voices in research and health policies decision making (Slejko et al. 2019), enabling more patient-centered care. Our study finds evidence that the general population cares about both bleeding gums on brushing and aesthetics but values the latter more highly. It is therefore important that service providers and decision makers consider both the potential health and nonhealth benefits of services when making resource allocation decisions.

The DCE also has some limitations. The general population clearly places a high value on dental services (SP, OHA), even when controlling for bleeding and aesthetic outcomes. While source of value to individuals is not relevant when one aims to maximize societal well-being, policy makers may legitimately require a deeper understanding of the reasons for these values before they can have confidence in the validity of the results and use them to inform dental health care policy. There are several potential explanations underpinning the high service valuation. Respondents may have valued the interaction with their dentist or hygienist, which provides an opportunity to identify any dental health issues and provide reassurance. Respondents may have valued SP because it is something they have always had (habitual) and have been encouraged to have from their dental health professionals (supplier-induced demand). Respondents may also be communicating an option value to have SP or detailed OHA. Further qualitative research is required to elaborate on the reasons behind the high valuation of SP.

## Conclusion

The UK general population place a high value on routinely provided primary dental care services, especially scale and polish. It is important that health care policy makers consider all sources of value when making resource allocation decisions, including service redesign. These include the preferences of service users, as well as the evidence regarding health benefits. Further research is required to provide additional insights into the reasons why people place such a high valuation on these services.

## Author Contributions

D. Boyers, contributed to conception, design, data acquisition, analysis, and interpretation, drafted the manuscript; M. van der Pol, V. Watson, contributed to conception, design, data acquisition, analysis, and interpretation, critically revised the manuscript; T. Lamont, A. Duncan, contributed to design, critically revised the manuscript; B. Goulao, contributed to design and data interpretation, critically revised the manuscript; C. Ramsay, contributed to conception and design, critically revised the manuscript; L. Macpherson, contributed to data interpretation, critically revised the manuscript; J. Clarkson, contributed to conception, design, and data interpretation, critically revised the manuscript. All authors gave final approval and agree to be accountable for all aspects of the work.

## Supplemental Material

sj-pdf-1-jdr-10.1177_0022034521989943 – Supplemental material for The Value of Preventative Dental Care: A Discrete-Choice Experiment Click here for additional data file. Supplemental material, sj-pdf-1-jdr-10.1177_0022034521989943 for The Value of Preventative Dental Care: A Discrete-Choice Experiment by D. Boyers, M. van der Pol, V. Watson, T. Lamont, B. Goulao, C. Ramsay, A. Duncan, L. Macpherson and J. Clarkson in Journal of Dental Research
