# Supplementary material for: The Value of Preventative Dental Care: A Discrete-Choice Experiment
Source: J Dent Res. 2021 Feb 4;100(7):723–30. doi: 10.1177/0022034521989943 (PMC8217903; doi:10.1177/0022034521989943)
Supplement: sj-pdf-1-jdr-10.1177_0022034521989943 – Supplemental material for The Value of Preventative Dental Care: A Discrete-Choice Experiment [file sj-pdf-1-jdr-10.1177_0022034521989943.pdf]

Appendix

Title: The Value of Preventative Dental Care: A Discrete Choice Experiment

Authors: Boyers, D.[1], van der Pol, M.[1], Watson, V.[1], Lamont, T.[2], Goulao, B[3] Ramsay, C.[3], Duncan, A.[3], Macpherson, L.[2], Clarkson, J.[2]

[1] Health Economics Research Unit, University of Aberdeen, Aberdeen, UK

[2] School of Dentistry, University of Dundee, Dundee, UK

[3] Health Services Research Unit, University of Aberdeen, Aberdeen, UK

Corresponding author: Dwayne Boyers, Health Economics Research Unit, University of Aberdeen, Polwarth building, Foresterhill, Aberdeen, AB25 2ZD; email: d.boyers@abdn.ac.uk

Background work to inform DCE design

The DCE design approach followed an iterative process of literature reviews, focus group research and think aloud studies to ensure the questions posed were realistic to respondents. Appendix Appendix Figure 1 describes the five-stage DCE design process (Ryan, et al., 2008).

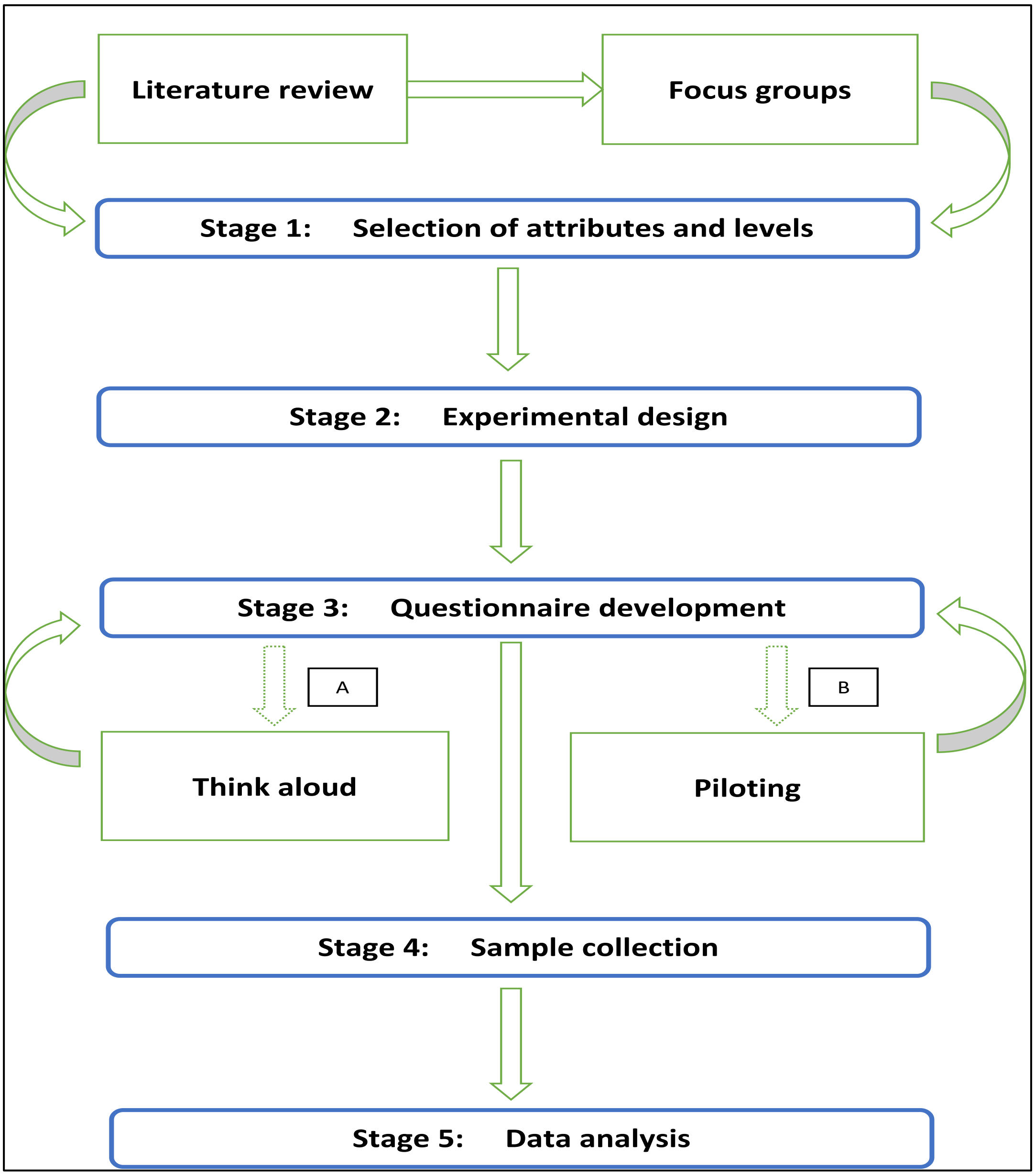

Note: Blue boxes indicate the five different stages of a DCE development, design and analysis. Green boxes indicate the methodologies that contribute to the development of stages 1 and 3. Within Stage 3, the questionnaire was first evaluated using "Think Aloud" methods (Box A), revised and then piloted with a soft launch (Box B).

Appendix Figure 1 Overview of DCE development process

Literature Review

Literature Review Methods:

A structured literature search was conducted to identify outcomes of dental care that might be included as attributes in the DCE. Medline and Embase databases were searched for English language studies, published between 2000 and 2014. A full search strategy is provided in Appendix Appendix Table ***1***.

Appendix Table 1 Medline and Embase search strategy

1. *dentistry/
2. (oral adj1 (health$ or hygiene$ or care$)).mp. [mp=ti, ab, sh, hw, tn, ot, dm, mf, dv, kw, nm, kf, px, rx, an, ui]
3. (gingival adj1 bleed$).tw.
4. (bleed$ adj1 gum$).tw.
5. periodontal disease.tw.
6. (dent$ or dont$).ti,ab.
7. or/1-6
8. ((patient$ or public$ or population$ or person$) adj3 (prefer$ or attitude$ or opinion$ or experience$)).ti,ab.
9. qualitative research/
10. (qualitative or interview$ or focus group$ or questionnaire$ or survey$).tw.
11. 9 or 10
12. 7 and 8 and 11
13. limit 12 to yr="2005 -Current"
14. limit 13 to animal studies
15. limit 14 to animals
16. 14 not 15
17. limit 16 to cancer
18. 16 not 17
19. limit 18 to english language
20. remove duplicates from 19.

The search included qualitative studies, and surveys of preferences, experiences, attitudes, beliefs or opinions of adult patients or the adult general population (mean / median age 18 and over) relating to routine dental care. Studies exploring preferences of participants with major dental problems including those requiring dental implants or those problems related to other significant diseases, such

as oral cancer, diabetes and HIV were excluded as they were not relevant to the research question which focussed on preventative care delivered in routine primary dental practice.

Literature Review results:

N=381 potentially relevant titles were retrieved. After an initial screen of titles and abstracts against the inclusion / exclusion criteria, N=21 potentially relevant full text articles were retrieved for further assessment. N=6 articles were excluded after full text assessment. The remaining N=15 studies were included in the review, and narratively synthesised to identify common themes and issues of importance to participants when attending routine dental care. A summary of the findings of each of the included studies from the review is provided in Appendix Appendix Table **2**.

The review finds that dental patients' value (i) continuity of care, because always seeing the same dental professional instils confidence (Dyer, et al., 2013; Sonneveld, et al., 2013; Sonneveld, et al., 2011) and (ii) being treated as a person rather than a patient (Sbaraini, et al., 2012), based on having a good relationship with dental care providers and meaningful input into the care they receive (Stenman, et al., 2009; Luzzi & Spencer, 2008). Fear of attending the dentist was identified as a significant barrier to more regular attendance for dental care (Gussy, et al., 2013; Guzeldemir, et al., 2008).

Provider of care (dentist, technician or hygienist) impacts on perceived quality of care received (Ohrn, et al., 2008; Dyer, et al., 2013). The cost of care, and frustrations about cost can impact on attendance for dental care (Dyer, et al., 2013; Stenman, et al., 2009; Mussard, et al., 2008). Additionally, costs may act as a signal of care quality, with potential differences in how much one would be willing to pay for care provided by dentists and hygienists (Dyer, et al., 2013).

The literature review shows that patients value the process and organisational aspects of care. Scale and polish services are positively perceived by dental attenders, even if they are sometimes uncomfortable to receive (Bonner, et al. 2005; Jones et al., 2013). One study shows that UK dental attenders without severe periodontitis have a strong belief that scale and polish is beneficial in terms of maintaining healthy gums, preventing bad breath, improving aesthetic outcomes (white teeth), and in preventing tooth decay (Jones et al., 2013).

However, there is little evidence about preferences for dental health outcomes. Studies show that respondents are willing and able to provide information on bleeding gums, but no information exists on when patient's view bleeding as an important outcome that would, for example, trigger a visit to the dentist (Stenman, et al., 2009; Fukai, et al., 2012; Bonner, et al., 2005).

Appendix Table 2 Summary findings of literature review

Study, (Year)	Study Objective	Population / setting	Country	Study design / research method used	N	Results / Themes / issues of importance
Dyer, et al., (2013)	To determine what matters to patients when their care is delegated to dental therapists	Purposive sample of patients in primary dental care.	UK	Semi structured in depth interviews	15	Patients reported positive experiences of treatment by dental therapists.<br><br>Two themes emerged as being important: (i) perceptions of the nature of dental services (dental team hierarchy – rationale for skill mix, preferences for having a choice of provider and also the cost) and (ii) trust and familiarity in the dental team (affective behaviour, communication and continuity of care).<br><br>Some insights related to linking cost to provider (e.g. hygienist may be quicker and thus cheaper) – an expectation that skill mix impacts cost, so hygienist may be cheaper as less qualified.
Jones, et al., (2013)	To investigate patient perceptions of the benefits of single visit scale and polish.	Non severe periodontitis dental attenders.	UK	RCT (comparing 6, 12, 24 month scale and polish), patient questionnaires	369	Responses support the opinion among patients that scale and polish will keep gums health, stop decay, improve feeling of mouth and improve appearance.<br><br>Most felt that scale and polish was important for oral health and for mouths to be aesthetically and socially acceptable.<br><br>Overall, there was a strong belief at baseline about the benefits of scale and polish for many things (e.g. healthy gums (80%), prevent bad breath (50%), whiten teeth (50%), and prevent tooth decay (80%). Positive views were stronger at follow up (with experience of regular scale and polish).
Hayes, et al., (2013)	To explore oral health	Non-fee paying dental	Ireland	Patient questionnaires	254	The majority of patients preferred having fillings over tooth extractions. There was a distinct preference for keeping ones teeth.

Study, (Year)	Study Objective	Population / setting	Country	Study design / research method used	N	Results / Themes / issues of importance
	attitudes of patients	practice attendees.		administered in dental waiting room		Regular attendance had a large impact on whether patients were dentate or edentate. Dentate patients were more regular attenders. Many edentate patients were only emergency attenders.
Gussy, et al., (2013)	To review the literature on qualitative research in dentistry.	Multiple populations and settings in review	N/A	Systematic review	n/a	Common qualitative methods in dentistry were interviews and focus groups. Little work on specific dental conditions.<br><br>Important issues relating to perceptions of general dental health were (i) water fluoridation; (ii) Dental services provision; (iii) dental health related quality of life. Anxiety was important.
Sonneveld, et al., (2011)	To explore the most important organisational aspects of a general dental practice.	Broad range of dental patients	Netherlands	Postal questionnaire	5000	Most important aspects were (50% or more stated in top 10 most important issues): Telephone access to dentist, ongoing education of dentist, Dutch speaking, waiting times in office, provision of information on treatments, waiting list for appointment.<br><br>Other issues of importance: (continuity of care, specialist available, professional standards, information on bill, reminder of recall, evening / weekend opening, accessibility).
Fukai, et al., (2012)	To investigate relationship between preferences, educational intervention and dental check-up	General dental patients (workplace interventions)	Japan	Questionnaire	204	Bleeding gums was the only aspect to change preferences due to the workplace delivered intervention.<br><br>Adds a degree of confidence that patients will place value on bleeding gums.<br><br>The study suggests it is probably feasible to ask patients about preferences for bleeding gums.
Sonneveld, et al., (2013)	To compare patient vs. dentists views of dental care organisational	General dental practices	Netherlands	Questionnaire	3127	Limited agreement on issues of importance between patients and providers.<br><br>Issues of importance to patients include: native language, wait times, continuity of care, treatment information on bill, fast emergency care.

Study, (Year)	Study Objective	Population / setting	Country	Study design / research method used	N	Results / Themes / issues of importance
Sbaraini, et al., (2012)	To explore the relationship between patient and dentist for preventative dental care.	Regular dental patients	Australia	Patient interviews	17	Patient expectations based more on attitudes and communication skills of dentists than technical competence. Patients valued a confident dentist who explains, listens and is friendly.<br><br>Within dental care setting: patients valued: a caring dentist; respect; listens; doesn't blame for poor oral hygiene.<br><br>Having a structured preventative approach in place was important. Patients were more likely to comply if they were treated as a person rather than a patient.<br><br>Patients valued a caring dentist, support, reassurance, dedicated dental team. Important issues for adherence to preventative care.
Stenman, et al., (2009)	To analyse patient attitudes to oral health and experiences of periodontal treatment	Dental patients	Sweden	Open ended interviews, using grounded theory. Data analysed in a hierarchical coding process	16	This core concept of importance was gaining a better understanding of dental disease and was related to 4 dimensions / categories:<br>- The need to be treated respectfully<br>- Gaining insight<br>- Frustration about treatment cost<br>- Feeling of control over the situation<br><br>Conclusion – patient communication is important, especially to explain complex issues of cost, and the impact of bleeding gums on future disease risk
Furnham & Swami, (2009)	To explore preferences for dentists	Dental Patients	UK	Questionnaire (online)	257	Two main effects were indicated.<br>- Preference for younger dentists<br>- Preference for dentists trained in Britain
Guzeldemir, et al., (2008)	To evaluate link between pain and dental anxiety	Dental patients	Turkey	Questionnaire	113	Fear, pain and anxiety are common and important barriers to attending scaling treatment.

**Study, (Year)**	**Study Objective**	**Population / setting**	**Country**	**Study design / research method used**	**N**	**Results / Themes / issues of importance**
	in scale and polish					
Ohrn, et al., (2008)	To determine if attitudes towards dentists and hygienists differ	Dental patients	Sweden	Questionnaire	364	Less negative attitude towards the hygienist vs. dentist (difference most pronounced for young people, least pronounced for those with periodontal disease).<br><br>There may be important differences between perceptions of care provided by hygienists or dentists.
Luzzi & Spencer, (2008)	To examine association between attitudes of service users and intention to visit dentist	Dental service users	Australia	Questionnaire, analysis based on the theory of planned behaviour.	517	Positive attitudes to dentist; perceived lack of control over visiting.<br><br>Attitudes, subjective norms, self-efficacy and perceived control were significant predictors of intention to attend.<br><br>Intentions, self-efficacy and past attendance (building up a habitual behaviour over time) were significant predictors of actual attendance.<br><br>Clinical attributes (e.g. tooth loss) were significantly correlated with intention to attend dentist. Process attributes (e.g. waiting lists) were not.
Mussard, et al., (2008)	To develop a patient experience questionnaire	Dental service users	UK (Croydon PCT)	Questionnaire development informed by literature review.	N/A	Literature review identified 5 generic dimensions of patient satisfaction with dental care:<br>○ technical competence,<br>○ interpersonal factors;<br>○ convenience;<br>○ costs;<br>○ experience<br>There were differences in satisfaction by age and ethnic grouping and those attending with pain / emergency were generally less satisfied.

Study, (Year)	Study Objective	Population / setting	Country	Study design / research method used	N	Results / Themes / issues of importance
Bonner, et al., (2005)	To investigate attitudes towards the benefits and costs of a simple scale and polish.	Dental service users	UK (Scotland)	Questionnaire developed to explore attitudes alongside a RCT of manual vs ultrasonic scale and polish.	420	99% believed scale and polish was beneficial, but most felt at least some discomfort when having the treatment.<br><br>Reasons for patients having a scale and polish were: tartar (46%); stained teeth (36%); bleeding gums (20%); mouth felt unclean (16%), gum disease (13).<br><br>44% answered that they always have a scale and polish with a check-up – for 22% this was the only reason given, suggesting that the scale and polish is part of a routine & could be supplier driven.<br><br>Study shows that patients view scale and polish positively, despite experience some discomfort with the procedure.<br><br>*Other points: (i)* majority of patients in Scotland were unaware of the correct charge for PI. (ii) Patients and providers both had very positive attitudes towards scale and polish.

N/A: Not Applicable; **PCT:** Primary Care Trust; **PI:** Periodontal Instrumentation (Scale and Polish); **RCT:** Randomised Controlled Trial

Literature Review References

Bonner, B. et al., 2005. A randomised controlled trial to explore attitudes to routine scale and polish and compare manual versus ultrasonic scaling in the general dental service in Scotland. *BMC Oral Health,* 5(3).

Dyer, T., Owens, J. & Robinson, P., 2013. What matters to patients when their care is delegated to dental therapists?. *Br Dent J,* 214(6), p. E17.

Fukai, K., Yoshino, K., Ohyama, A. & Takaesu, Y., 2012. Dental patient preferences and choice in clinical decision making. *Bull Tokyo Dent Coll,* 53(2), pp. 59-66.

Furnham, A. & Swami, V., 2009. Patient preferences for dentists. *Psychol Health Med.,* 14(2), pp. 143-149.

Gussy, M., Dickson-Swift, V. & Adams, J., 2013. A scoping review of qualitative research in peer reviewed dental publications. *Int J Dent Hygiene,* Volume 11, pp. 174-179.

Guzeldemir, E., Toygar, H. & Cilasun, U., 2008. Pain perception and anxiety during scaling in periodontally health subjects. *J periodontol,* 79(12), pp. 2247-2255.

Hayes, M. et al., 2013. An analysis of the attitudes of dental patients attending general dental practice in Galway.. *J Ir Dent Assoc,* 59(4), pp. 179-82.

Jones, C. et al., 2013. Patient perceptions regarding benefits of single visit scale and polish: a randomised controlled trial. *BMC Oral Health,* 13(50).

Luzzi, L. & Spencer, A., 2008. Factors influencing the use of puiblic dental services: An application of the theory of planned behaviour. *BMC Health Services Research,* Volume 8, p. 93.

Mussard, J. et al., 2008. What do you think of your dentist? A dental practice assessment questionnaire. *Journal of evaluation in clinical practice,* Volume 14, pp. 181-184.

Ohrn, K., Hakeberg, M. & Abrahamsson, K., 2008. Dental beliefs, patients' specific attitudes towards dentists and dental hygienists: a comparative study. *Int J Dent Hygiene,* Volume 6, pp. 205-213.

Sbaraini, A., Carter, S., Evans, R. & Blinkhorn, A., 2012. Experiences of dental care: what do patients value?. *BMC Health Services Research,* Volume 12, p. 177.

Sonneveld, R. et al., 2013. Patients' priorities in assessing organisational aspects of a general dental practice. *International Dental Journal,* Volume 63, pp. 30-38.

Sonneveld, R. et al., 2011. The estimation of patients' views on organizational aspects of a general dental practice by general dental practitioners: a survey study. *BMC Health Services Research,* Volume 11, p. 263.

Stenman, J., Hallberg, U., Wennstrom, J. & Abrahamsson, K., 2009. Patients' attitudes towards oral health and experiences of periodontal treatment: A qualitative interview study. *Oral Health Prev Dent,* Volume 7, pp. 393-401.

Focus groups

Focus Groups (FGs) methods

Additional primary focus group (FG) research was conducted to better understand how people value dental health outcomes, and to ensure that the most meaningful attributes and levels were included in the DCEs. FGs were conducted with members of the general population in Aberdeen, Scotland in 2015. Discussions followed a pre-specified topic guide, designed with the intention of determining A) preventative dental care services that were of value to service users', and included specific prompts to gain an understanding of respondents experiences of scale and polish and oral hygiene advice (OHA) in particular; B) the outcomes of preventative dental care that were of most value to respondents, with the intention of identifying outcomes for inclusion in the DCE. Specific prompts were included to elicit information on bleeding gums if this was not brought up naturally by participants in the groups. Further details are provided in the topic guide. Discussions were led by DB with assistance from VW who took notes. Respondents were compensated with £10 'love to shop' vouchers at the end of the discussions.

Eighteen respondents were recruited into four FG sessions, with four or five respondents per group. FGs were recorded and transcribed. Three researchers independently read the transcripts and analysed the data using a qualitative thematic framework, using colour coding to highlight different emergent themes from the FG data. The researchers then met to agree on the set of themes that might be included as potential attributes in the DCE. Each attendee at the meeting independently presented their set of lists of themes as coded from the data, and these were drawn on a flip-chart, first identifying the themes that were common across all researchers and supplementing this with additional themes and sub-themes presented by a researcher and agreed based on consensus and discussion among the group. The outcome of the meeting was a full set of themes, processes and treatments / outcomes on a single flip chart diagram, with thematic, process and outcome links identified.

Participants also completed a short questionnaire after the group discussions. The questionnaire collected demographic information to ensure a rich data set of views and opinions, and a payment card willingness to pay (WTP) question to help set the levels of the cost attribute for the DCE. The FG study protocol was approved by the University of Aberdeen College Ethics Review Board (CERB: 2015/1/1170).

Focus Groups (FGs) results:

The FGs provided a rich dataset and participants had varied demographic characteristics. FG participants completed two payment card questions stating their maximum WTP for a scale and polish, and personalised OHA. Respondents to the supplementary FG questionnaire stated a WTP range of £10 to £50 and £0 to £90 for a scale and polish and personalised OHA respectively. Saturation was achieved after the 4th FG with no new themes emerging in either FG3 or FG4. A schematic of the findings from a thematic analysis of FG data is presented in Appendix Appendix Figure **2**.

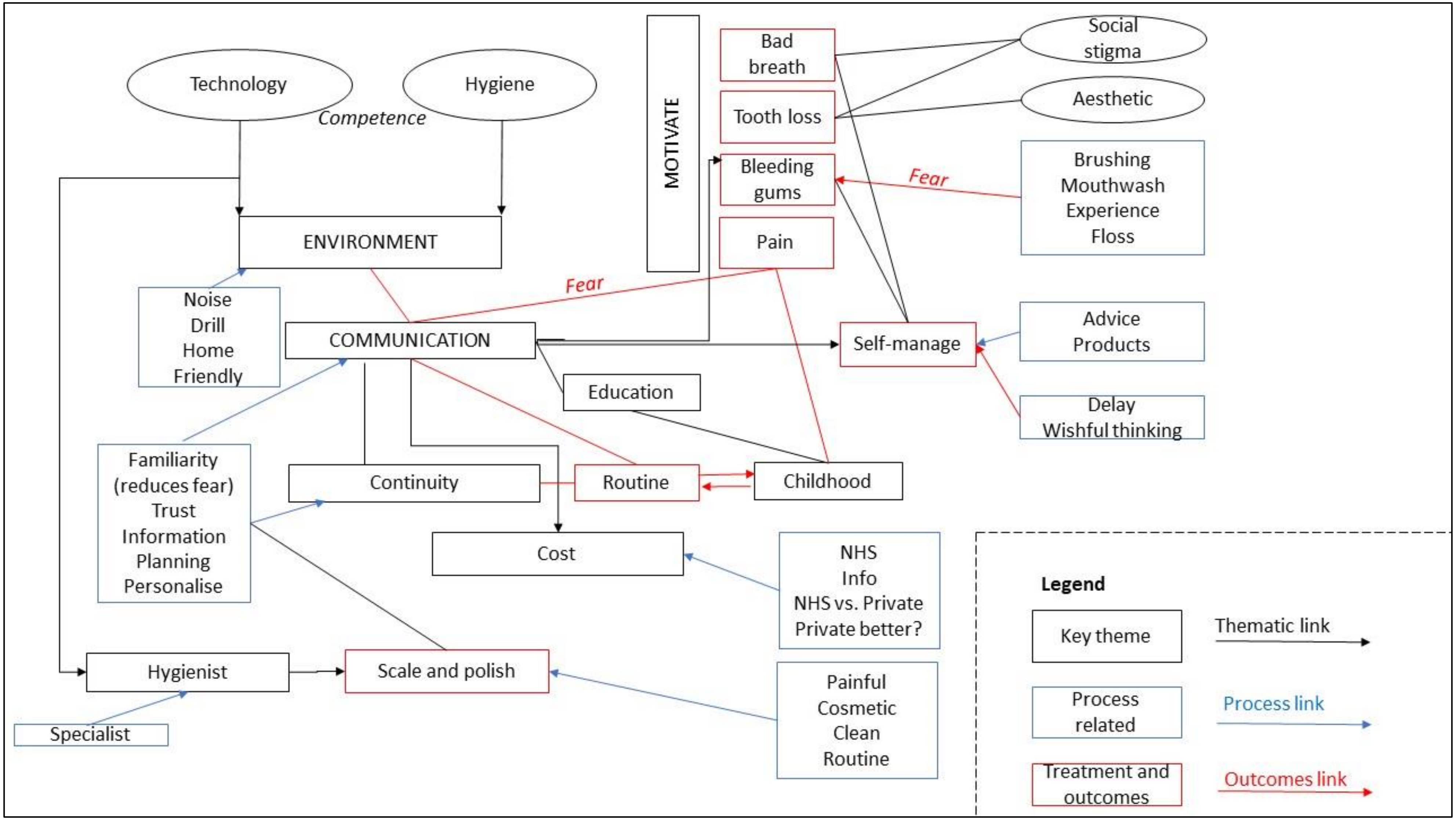

Appendix Figure 2 Schematic of Focus Group Results

Participants valued dental care services such as personalised oral hygiene advice and scale and polish. Personalised care was generally preferred to generic or routine care, but the meaning of 'personalised' was individual specific. The respondents understanding of "personalised" varied widely, from a brief discussion to detailed advice with a personalised action plan on how to improve self-care. Therefore, the DCE included additional descriptive text to specify the exact meaning of "personalised" (as per the context of the IQuaD study) to ensure the valuations provided by the DCE respondents were congruent with the service being evaluated.

The dental professional providing scale and polish and oral hygiene advice services was important. Many participants were unaware of the dental hygienist's role, and knowledge of this was dependent on whether they had ever experienced treatment from a hygienist, and whether it was a positive or negative experience. In general, participants who had previously received treatment from hygienists felt that the quality of the care they received was good, with some respondents describing the hygienist as a "specialist role", in which excellent care could be delivered. However, others felt that the hygienist role was limited, and they had more confidence that a dentist would provide better quality of care overall. Some respondents linked this to their willingness to pay and felt they would pay more for care from a dentist, and that this would be acceptable to them due to perceived better quality of care.

The frequency of recall for attendance at the dental practice was important to respondents, and many considered having a scale and polish to form an integral component of attending for a check-up. With regards to preventative dental care outcomes, participants preferred to avoid dental pain, tooth loss, and bad breath. There was consensus across respondents that avoidance of pain was the most important outcome from dental care. Pain was viewed as a cause for concern, an indication that something was wrong, and was a trigger for most participants to seek emergency dental care. People with experience of dental decay had a strong preference to avoid any stage of dental decay, even if it was non-symptomatic.

Avoidance of bleeding gums was also important to FG participants. However, in two groups, this only emerged after probing by the facilitator. Bleeding gums were associated with brushing, and those with more frequently bleeding gum were generally less bothered by it. Aesthetic outcomes were considered important with many respondents describing how they liked having scale and polish because it made their teeth look and feel clean and healthy.

The FG work was instrumental in arriving at a set of attributes and levels for inclusion in the DCE. The following decisions were made regarding the attributes and levels to be included in the DCEs based on the FG work:

- Scale and polish and oral hygiene advice were included as attributes to value the IQuaD study interventions. However, provider levels (dentist or hygienist) were added to each attribute to reflect potentially important implications of care provider on service preferences.

- Whilst pain (from caries / decay) was an important, and dominant outcome from the FGs, it was not included in the DCE because there was no clinical or patient experience evidence linking it to the scale and polish intervention, and its inclusion would likely dominate other important outcome attributes to be valued for the study.

- The FG work clearly identified that respondents would not be particularly concerned by some bleeding at the dental practice. In fact, many expected it. Therefore, in the DCE, bleeding gums was set in the context of bleeding when brushing teeth to make this attribute more meaningful to respondents, reflecting the FG discussions.

- Participants highly valued the aesthetic benefit of having a scale and polish. An aesthetic attribute, with levels of how clean respondents' teeth look and feel was therefore added to the DCE with the intention of valuing wider, non-health, benefits of scale and polish or oral hygiene advice.

- The FG provided valuable information to help contextualise and describe attribute levels in the DCE. For example, detailed descriptions of hygienists, and personalised OHA were provided in the survey material based on ambiguities and uncertainties identified in the FGs. Particular effort was made in the DCE descriptive text to dis-entangle the value associated with scale and polish, from having a dental check-up based on the links identified in the FGs.

There was no evidence from the FG work to suggest any further important interactions between the different attributes that emerged from the FGs that would were ultimately included in the DCE (for example, service provider and outcome). It was therefore deemed appropriate that the DCE should follow a main effects only design.

Focus group topic guide

<u>Topic guide for conduct of the focus groups in dental care:</u>
(For researcher use only):
Total time for Focus Group (90 mins)

<u>Registration / tea / coffee</u>
(10 mins)
Registration, seating, collection of consent forms, welcome participants individually, get participants to fill in a short demographic questionnaire and fill in any other attendance paperwork necessary.

<u>Opening round</u>
(10 mins)
Introduction of researchers and short summary of the project *(without expanding on the nature of the topics to follow in discussions)*

Get participants to give their name and to tell the group something about them *(to get people introduced to each other)*. Researcher / facilitator to start this off.

<u>Experiences of dental care:</u>
(10 mins)
I want to start by thinking about the last time that you attended your dental practice. Don't worry if you haven't been for a while or if you've never been at all. You can just think about what you would expect a visit to the dentist to be like. This could be a visit to your dentist, your hygienist or another dental assistant.

1. <u>Can you please tell me a bit about what parts of the experience you liked or enjoyed, and what parts you found difficult or challenging?</u>
   Prompt:
   - *Do you attend regularly / less frequently – what makes you attend your dentist more / less often*
   - *Why do you go to your dentist? (Is it because you want to or because you feel you have to? – is it that your dentist has good facilities…?*
   - *What did you get out of your last dental visit, or what did you enjoy about the overall experience?*
   - *How often do you go to your dentist / how often do you feel you should go? What would encourage you to go more often?*

<u>Attitudes to and experiences of dental care in general and good oral health:</u> (15 mins – 5 mins per question)
Brainstorm what 'good oral health' means to you.

2. <u>What is good oral health?</u>
   Prompt: Is it clean white teeth / is it bad breath / is it bleeding gums / is it more about the future – e.g. missing teeth or keeping your own teeth.
3. <u>(If not brought up naturally in the discussion): How important do you think bleeding gums are?</u>
   Prompt: What is important about bleeding gums: is it what others might think / is it that they are unsightly / is it that they may lead to long term oral health problems?
4. <u>When you think of bleeding gums, how would you describe it?</u>
   Prompt: Is it when you bleed when you brush your teeth?
   Does it matter how many teeth are bleeding or is it to do with the overall amount of blood when you spit when brushing?

<u>Attitudes towards ways to achieve "Better oral health"</u>
(25 mins – 5 mins per topic)

Up until now we have discussed some general issues of what oral health is, how we identify poor oral health and what we think the important outcomes of good oral health care. Now in this section, we want to focus a little more closely on some of the common ways in which we might achieve better oral health, and what our dental care professionals can do to help. A number of ways have been suggested to help.

5. <u>How can you achieve good oral health?</u>

Prompt:
- Is it more frequent dental visits / scale and polish / personalised hygiene advice?
- How would you identify poor oral health / how would you know something wasn't quite right and you might need to go to the dentist?

If not brought up directly above, prompt specifically for the following:

One method suggested to improve oral health is how often you receive <u>*routine dental check-ups*</u>*, (so when you go to the dentist and receive an appointment for the next time, or a date to call and make an appointment).*

6. <u>What are the benefits to you of attending for regular routine dental check-ups?</u>

Prompt:

- How often would you like to / think you should see the dentist for a check-up?
- What would encourage you to attend your assigned recall more readily?
- What prevents you from attending at your regularly assigned time (e.g. 1 year) for a check-up?

The second way is to have a <u>*scale and polish*</u>*: If necessary, explain to participants what a scale and polish is at this point. Prepare some script to do this.*

7. <u>What do you think the reasons are for having a scale and polish? / What are the benefits?</u>

Prompt:

- How often would you like to / do you think you should have a scale and polish?
- What would encourage you to have a scale and polish?
- What would prevent you from having a scale and polish?

The third way is through oral hygiene advice provided by a dentist or hygienist.

8. <u>What does oral hygiene advice mean to you?</u>

Prompt:

- How should your dentist / hygienist help you maintain good oral health?
- Do you currently feel your dentist gives you enough support to maintain good oral health?
- Could they do more? If so, how could they do more?
- What are the benefits of good oral hygiene advice?

A specific type of <u>*personalised oral hygiene advice*</u> *has been suggested as being helpful: (Describe to participants what exactly personalised oral hygiene advice is, and how it may differ from routine oral hygiene advice).*

9. <u>Tell us how you feel about personalised oral hygiene advice, as we have described it to you?</u>

Prompt:

- Do you feel that personalised oral hygiene advice is a good idea?
- What do you like about it?
- What do you dislike?

<u>Closing Questions</u>

(10 mins)

Thinking about the issues we have discussed in this group (frequency of dental attendance, scale and polish). Think about someone who does not attend the dentist / or who is not a regular attendee for dental care. Imagine you had to give that person one piece of advice about the benefits of going to the dentist / having a scale and polish.

10. <u>What would you advise them as being the most important reason to attend regular check-ups?</u>

11. <u>Thinking about the same question, what advice would you give to someone who was interested in scale and polish?</u>

<u>Wrap up and "Thank You"</u>

(10 mins)

Give participants final contingent valuation willingness to pay question. Thank you very much for your time – encourage participants to collect their vouchers on the way out and to hand up the brief questionnaires they filled in at the start and end of the focus group.

Questionnaires provided to focus group respondents

SHORT QUESTIONNAIRE

(Provided before FG discussions commence):

What age are you? ☐ *Prefer not to answer (✓)* ☐

Question	Option	*Please tick (✓)*
**What is your gender?**	Male	
	Female	
	Other	
	Prefer not to answer	
**What is your current employment status?**	Employed full time	
	Employed part time	
	Self-employed / Independent professional / contractor	
	Student	
	Homemaker	
	Unemployed / seeking work	
	Retired	
	Other	
	Prefer not to answer	
**Which of the following qualifications do you have? Please tick all that apply**	Primary School Level	
	High school / Secondary School Level	
	Apprenticeship (e.g. electrician / plumber)	
	College or equivalent (e.g. diploma / advanced diploma)	
	University (degree or higher degree – e.g. BA, MA, PhD)	
	Professional qualification (e/g nursing accountancy)	
	Other vocational / work – related qualifications	
	Foreign Qualifications	
	No Qualifications	
**Please tell us about how you normally pay for dental care**	The NHS pays some of the cost & I also pay some	
	The NHS covers the full cost (I do not pay anything)	
	I pay the full cost directly (private care)	
	I have a dental payment plan agreed with my dentist	
	I am covered by dental insurance	
**How would you say your oral and dental health is in general?**	Very good	
	Good	
	Fair	
	Bad	
	Very bad	

**Do you regularly attend a dentist?**	Yes	☐
	No	☐
**When was the last time you attended the dentist?**	Under 6 months	☐
	6-12 months	☐
	12-24 months	☐
	Over 24 months	☐
	I have never attended the dentist	☐
**When was the last time you received a scale and polish?**	Under 6 months	☐
	6-12 months	☐
	12-24 months	☐
	Over 24 months	☐
	I have never received a scale and polish	☐
**When was the last time you were given advice by your dentist or hygienist on how to brush, floss or care for your teeth?**	Under 6 months	☐
	6-12 months	☐
	12-24 months	☐
	Over 24 months	☐
	I have never been given this advice	☐
**What is the most important reason for attending the dentist?**		
**What would you say is the most likely reason for not seeing your dentist?**		

HOW MUCH DO YOU VALUE DENTAL TREATMENTS?

(Questionnaire provided after conclusion of FG discussions)

We are interested in how much value you place on the different treatments we have discussed today. We are aware that many treatments you receive may be free of charge or the NHS may pay part of the cost. However, please imagine you have to pay the full price of the following treatments. Please tick the appropriate box to tell us if you would be willing to pay the amount of money shown for each of the three treatments.

Please answer the question below ticking either "**yes**" or "**no**" against **each cost** for the **three** treatments.

**Would you be willing to pay this amount for treatment?**	**A scale and polish provided by your dentist**		**Personalised oral hygiene advice, provided by your dentist**	
	**Yes**	**No**	**Yes**	**No**
**£0**	☐	☐	☐	☐
**£1**	☐	☐	☐	☐
**£5**	☐	☐	☐	☐
**£10.50**	☐	☐	☐	☐
**£15**	☐	☐	☐	☐
**£17.50**	☐	☐	☐	☐
**£20**	☐	☐	☐	☐
**£30**	☐	☐	☐	☐
**£50**	☐	☐	☐	☐
**£75**	☐	☐	☐	☐
**More than £75**	☐	☐	☐	☐
**If more than £75, please write the maximum amount you would pay**	£ .		£ .	

DCE design code

This section describes the NGENE software code used to generate the experimental design for the DCE survey. In brief, the code seeks to design a pivoted, segmented DCE design. There are three segments in the design (Good, Moderate, Poor dental outcomes) created according to respondent's current state of bleeding and aesthetic outcomes. The design then seeks to create an opt-out level that is specific to the design segment to improve realism of the choice tasks for respondents. The design then pivots the outcome levels around the reference alternative. The design code generates a main effects, d-efficient design to estimate a multinomial logit model of preferences.

Segment "Good"

```
Design
;alts = NONE, A*,B*
;rows = 30
;eff = (mnl,d)
;block = 3
;alg = mfederov
;reject:
A.BLEED=B.BLEED,
A.LOOK = B.LOOK
;model:
U(NONE) = b3[0]*BLEED.ref[3] + b4[0]*LOOK.ref[3]
/
U(A) = b0[0] + b1.effects[0|0]*Advice[1,2,3] + b2.effects[0|0|0|0]*PI[1,2,3,4,5] +
b3.effects[0.1|0.1]*BLEED.piv[-2,-1,0](8-12,8-12,8-12)+ b4.effects[-0.1|-0.1]*LOOK.piv[0,1,2](8-12,8-12,8-
12) + b5.effects[0|0|0|0]*COST[1,2,3,4,5]
/
U(B) = b0 + b1*Advice+ b2*PI + b3*BLEED.piv[-2,-1,0](8-12,8-12,8-12) + b4*LOOK.piv[0,1,2](8-12,8-12,8-
12) + b5*COST[1,2,3,4,5]
$
```

Segment "Moderate"

```
Design
;alts = NONE, A*,B*
;rows = 30
;eff = (mnl,d)
;block = 3
;alg = mfederov
;reject:
A.BLEED=B.BLEED,
A.LOOK = B.LOOK
;model:
U(NONE) = b3[0]*BLEED.ref[4] + b4[0]*LOOK.ref[2]
/
U(A) = b0[0] + b1.effects[0|0]*Advice[1,2,3] + b2.effects[0|0|0|0]*PI[1,2,3,4,5] +
b3.effects[0.1|0.1]*BLEED.piv[-2,-1,0](8-12,8-12,8-12)+ b4.effects[-0.1|-0.1]*LOOK.piv[0,1,2](8-12,8-12,8-
12) + b5.effects[0|0|0|0]*COST[1,2,3,4,5]
/
```

U(B) = b0 + b1*Advice+ b2*PI + b3*BLEED.piv[-2,-1,0](8-12,8-12,8-12) + b4*LOOK.piv[0,1,2](8-12,8-12,8-12) + b5*COST[1,2,3,4,5]

$

Segment “Poor”

```
Design
;alts = NONE, A*,B*
;rows = 30
;eff = (mnl,d)
;block = 3
;alg = mfederov
;reject:
A.BLEED=B.BLEED,
A.LOOK = B.LOOK
;model:
U(NONE) = b3[0]*BLEED.ref[5] + b4[0]*LOOK.ref[1]
/
U(A) = b0[0] + b1.effects[0|0]*Advice[1,2,3] + b2.effects[0|0|0|0]*PI[1,2,3,4,5] + b3.effects[0.1|0.1]*BLEED.piv[-2,-1,0](8-12,8-12,8-12)+ b4.effects[-0.1|-0.1]*LOOK.piv[0,1,2](8-12,8-12,8-12) + b5.effects[0|0|0|0]*COST[1,2,3,4,5]
/
U(B) = b0 + b1*Advice+ b2*PI + b3*BLEED.piv[-2,-1,0](8-12,8-12,8-12) + b4*LOOK.piv[0,1,2](8-12,8-12,8-12) + b5*COST[1,2,3,4,5]

$
```

Description of attributes and levels

Dental Hygienist:

A **dental hygienist** can help prevent and treat gum disease. Hygienists can give advice on how to achieve good oral health. This includes teaching you how to look after your teeth and gums. They can show you how to keep your teeth plaque free and how to prevent tooth decay.

A **dental hygienist** may give diet advice to help you maintain good oral health. Hygienists can professionally clean your teeth by giving you a 'scale and polish'. Hygienists do not give you advanced treatments, such as extracting adult teeth, giving root canal treatments, crowns or dentures.

Scale and Polish:

A scale and polish is a professional clean for your teeth. Regular scaling and polishing can help keep your teeth and gums healthy, clean and fresh.

Scaling removes the hard tartar which forms on your teeth like scale inside a kettle. It also removes trapped food and plaque.

Your dentist or hygienist will use a rotating brush or rubber polisher with toothpaste to **polish** your teeth. Polishing helps to clean stains off the surfaces of your teeth, helping them look and feel clean.

Oral Hygiene Advice

Oral hygiene advice is any type of advice about how to keep your teeth, gums and mouth clean, fresh and healthy. It might include:

- Telling or showing you on how to brush, floss and clean your teeth.
- Advising you when to brush and how often.
- Giving you information about how your diet can help with oral health.

Oral hygiene advice is normally provided during a routine check-up. It may be a short chat or a general discussion with your dentist or hygienist.

Detailed and Personalised Oral Hygiene Advice

Detailed and personalised oral hygiene advice

Researchers are looking at a new way in which dentists and hygienists could provide oral hygiene advice. This new approach is called **Detailed and personalised** oral hygiene advice.

Detailed and personalised advice is more detailed than what you might normally get at a dental check-up. It is designed just for you. Your dentist or hygienist will decide, with you, what your needs are.

You will have a separate or longer appointment with the dentist or hygienist where you get **All** of the following:

- Advice about how to take care of your teeth by yourself.
- Information on how to recognise if something is wrong.
- You will be taught and shown how to brush and floss properly.
- Your dentist / hygienist will agree a detailed action plan with you.
- Your dentist or hygienist will check on your progress at your next dental check-up.
- You will be given more advice if needed.

If you need other treatments (e.g. Scale and polish) after your advice, this will count as a new appointment. The new appointment could be on the same day, or you might be asked to come back another time.

Bleeding gums

Bleeding gums are often a sign of gum disease, also called gingivitis. Gingivitis is when your gums become inflamed, swell and bleed, most commonly because of a build-up of dental plaque. Bleeding gums may cause bad breath.

If bleeding gums are not treated, gum disease may get worse. You could develop a more serious gum disease called periodontitis. Periodontitis damages the tissues that hold your teeth in place. If left untreated, it can cause the loss of these tissues; your teeth may become loose and may eventually fall out.

Many people first notice that they have bleeding gums when brushing their teeth

Choice task instructions

SECTION 2: CHOOSING BETWEEN DIFFERENT DENTAL CARE PACKAGES

In this section, we want to know what would matter to you if you were choosing a dental care package for the next three years at your dental practice. The dental care packages you may choose differ in:

- **Who provides the care (Your dentist or hygienist?)**
- **How many scale and polishes you get per year**
- **If you get a detailed and personalised oral hygiene advice appointment**

By getting a treatment package, your dental health and appearance might differ in:

How clean your teeth look and feel

How often you have bleeding gums when you brush your teeth (3 years from now)

The dental care packages also differ in:

The cost of the package per year for the next three years.

Please consider if you would be able and willing to pay the cost of each package. Remember, if you spend money on dental care, you may have less money available to spend on other things, such as a meal in a restaurant or a trip to the cinema.

Dental care packages:

Please read all the dental care packages carefully and indicate which option you would choose. Some of the questions may seem similar, but each question is different. There are no right or wrong answers. It is your opinion that counts.

What's included in the dental care packages?

Please remember that when you pay for a dental care package, you can only get the services included in that package. Any other services, such as additional check-ups or treatments (e.g. fillings or extractions) are not covered by the dental care packages offered.

No dental care package:
If you do not like, or are not willing to pay for either of the packages in a question, you will have the option to choose "No Dental Care". By choosing "No Dental Care", you will <u>not</u> receive a detailed and personalised oral hygiene advice appointment or any scale and polish, but your dental health may get worse.

You will now see an example choice to get you started.

EXAMPLE CHOICE

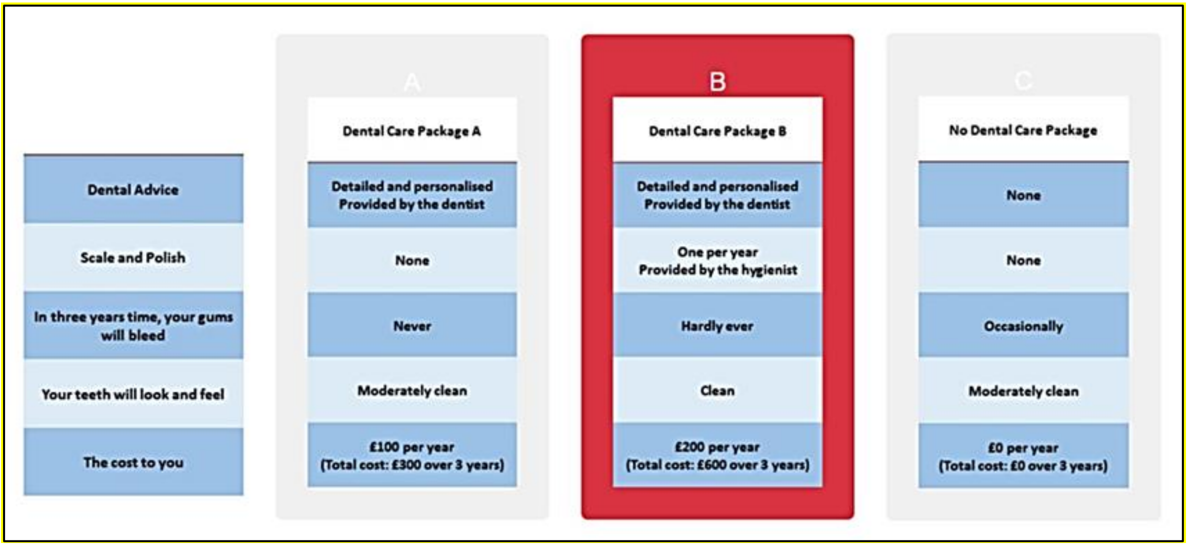

	A	B	C
	Dental Care Package A	Dental Care Package B	No Dental Care Package
Dental Advice	Detailed and personalised Provided by the dentist	Detailed and personalised Provided by the dentist	None
Scale and Polish	None	One per year Provided by the hygienist	None
In three years time, your gums will bleed	Never	Hardly ever	Occasionally
Your teeth will look and feel	Moderately clean	Clean	Moderately clean
The cost to you	£100 per year (Total cost: £300 over 3 years)	£200 per year (Total cost: £600 over 3 years)	£0 per year (Total cost: £0 over 3 years)

This person has chosen **Dental Care Package B.**

THINK ALOUD STUDY

The aim of the think aloud study was to test-run the draft design of the DCE to ensure that respondents understood the draft questions, traded attributes and levels as intended and to identify any bugs in the survey logic code.

Think aloud methods

There were two phases to the think aloud study. The first was conducted with five office colleagues. The second phase was conducted with ten members of the general population, in two rounds of five interviews, to enable incremental improvements to the survey design after each round. General population respondents were recruited through posters, mailing lists and gumtree adverts. Face to face interviews were conducted in 2016. Respondents were randomised (coin toss) to complete a draft version of the survey using A) a mouse or touchpad and B) completion in internet explorer or google chrome to explore ease of completion using different methods and interfaces. The purpose of this randomisation was to test ease of survey completion across a range of different interfaces and devices. The think aloud interviews were conducted by DB who also took notes.

Respondents were encouraged to continually verbalise their thoughts whilst completing the survey, and to give frank and honest opinions about the content and framing of the questions. When completing the choice tasks, respondents were encouraged to explain in detail how they made their choices, including which choice alternative they chose on each occasion and why. This was important to determine if respondents used any decision-making heuristics that could be problematic for the DCE analysis. Respondents were only interrupted during the process to encourage and remind them to verbalise their thoughts.

General population respondents were compensated for their time with £10 love to shop vouchers, and the discussions were audio recorded. Additional pre-planned de-briefing questions were posed after the respondent completed the survey to elaborate on any difficulties completing choice tasks, or any mis-understandings that arose.

Think aloud results

In general, respondents enjoyed answering the DCE questions, engaged well with the choice tasks and felt that the process of making trade-offs between the packages was appropriate and relevant. Some respondents found the process of making choices difficult, but trade-offs were made in the expected way comparing whole profiles of dental services against each other. Respondents also accepted the idea of paying for dental care, even if their maximum WTP was only equal to the lowest cost level (£10 per year), and no protest answers were identified.

Several minor issues were identified across the survey, due in part to the complexity of the logic code, and were corrected after each round of interviews. No new errors were identified in any of the final three interviews. One major issue was identified. Respondents in both surveys assumed that additional services would be provided at an appointment which were not described by the attribute levels. For example, two respondents assumed that they would receive oral hygiene advice alongside a scale and polish, even when the advice attribute stated "none". In three interviews, respondents felt that when receiving a scale and polish, they would also get a check-up and receive any treatment necessary.

Changes were therefore made to the wording of the respective attribute descriptors to clarify that we wished respondents to make choices based only on the attribute levels presented to them and to assume everything else was equal across groups and any other treatments (including check-ups) would have to be purchased out with the dental care packages offered. Respondents were also provided with additional guidance prior to commencing the choice tasks emphasising what was and was not included in dental care packages. These changes were made after the end of the first round of interviews and no new issues were identified in the final rounds (N=10). No changes were made to the DCE design at this stage as all attributes and levels were found to be acceptable, relevant and tradable to respondents.

SUBGROUP ANALYSES

Subgroup analysis methods

Pre-specified, planned subgroup analyses were conducted for participant characteristics by sex (male / female), UK region (Scotland / rest of UK), income (high / low), experience of scale and polish (yes/ no), and familiarity with dental hygienists (yes / no). The impact of subgroup membership on preferences was investigated using interaction terms between effects coded categorical variables and attribute level main effects. Models for each subgroup were compared to the base case model (without subgroup interactions) using likelihood ratio tests and effects are considered significant at $p<0.01$. For the purpose of investigating subgroup effects, the base case model does not apply survey probability weights for Scotland.

Subgroup analysis results

The likelihood ratio tests show no overall effect on preferences (jointly across all model parameters) for UK region or smoking status. For subgroups where the tests indicated an impact of subgroup on preferences, the following findings were observed.

- Respondents with experience of the hygienist valued the dental care packages more highly (disutility associated with cost attribute was lower). They also gained less utility from teeth that look and feel only moderately clean.
- The significant interaction effect on the Alternative Specific Constant (ASC) with experience of scale and polish indicates that those with experience of scale and polish are more likely to commit to a dental care package. They also valued the dental care packages more highly.
- Neither experience of seeing the dental hygienist or of having scale and polish impacted on the preferences of respondents for having these services, or for whether it is the dentist or hygienist who provides the service.
- Females in the sample gained greater utility from dental care packages where teeth look and feel clean or very clean. Similarly, they experienced greater dis-utility from teeth that look and feel moderately clean or unclean.
- As expected, the cost attribute had less of an impact on the preferences of higher income respondents.

The subgroup analyses indicate that whilst there were some differences across subgroups, in general the direction of attribute level effect remained similar with respondents preferring scale and polish and personalised advice, preferring less bleeding and teeth that look and feel clean and healthy as well as preferring lower cost dental care packages.

Appendix Table 3 Analysis of DCE subgroups

	Base case	Hygienist experience [a]	Country (Scotland) [b]	Sex (f) [c]	Income (£20,800+) [d]	Smoker (ever) [e]	Experience of Scale and Polish[f]
**Main effects**							
Personalised advice from Dentist	0.122 ***	0.133 ***	0.143 ***	0.124 ***	0.121 ***	0.140 ***	0.144 ***
Personalised advice from Hygienist	0.021	0.016	0.027	0.019	0.022	0.011	-0.027
12-m PI from Dentist	0.194 ***	0.199 ***	0.232 ***	0.194 ***	0.193 ***	0.208 ***	0.170 ***
12-m PI from Hygienist	-0.067 *	-0.065	-0.089 *	-0.071 *	-0.065 *	-0.100 **	-0.171 ***
6-m PI from Dentist	0.315 ***	0.309 ***	0.316 ***	0.323 ***	0.315 ***	0.319 ***	0.280 ***
6-m PI from Hygienist	0.279 ***	0.274 ***	0.281 ***	0.283 ***	0.277 ***	0.294 ***	0.261 ***
Bleeding gums – Hardly ever	0.307 ***	0.313 ***	0.337 ***	0.303 ***	0.312 ***	0.301 ***	0.280 ***
Bleeding gums – Occasionally	-0.046	-0.032	-0.094 **	-0.055	-0.043	-0.053	-0.143 **
Bleeding gums – Fairly often	-0.073	-0.058	-0.012	-0.073	-0.084	-0.055	-0.057
Bleeding gums – Very often	-0.572 ***	-0.599 ***	-0.591 ***	-0.556 ***	-0.573 ***	-0.608 ***	-0.429 **
Teeth look and feel – v. unclean	-0.899 ***	-0.989 ***	-0.887 ***	-0.845 ***	-0.868 ***	-0.901 ***	-0.719 ***
Teeth look and feel - unclean	-0.393 ***	-0.368 ***	-0.308 ***	-0.392 ***	-0.400 ***	-0.398 ***	-0.311 ***
Teeth look and feel – mod. clean	0.139 ***	0.183 ***	0.109 **	0.120 ***	0.134 ***	0.138 ***	0.049
Teeth look and feel - clean	0.514 ***	0.497 ***	0.448 ***	0.494 ***	0.508 ***	0.506 ***	0.402 ***
Annual cost	-0.011 ***	-0.011 ***	-0.011 ***	-0.011 ***	-0.011 ***	-0.011 ***	-0.012 ***
ASC *(Mean)*	0.465 ***	0.402 ***	0.448 ***	0.459 ***	0.472 ***	0.417 ***	0.239 **
(sd)	1.470 ***	1.435 ***	1.476 ***	1.473 ***	1.459 ***	1.464 ***	1.420 ***
**Interaction terms for subgroup with main attribute level effects**							
Personalised advice from Dentist		-0.034	0.032	0.011	-0.018	0.044	-0.023
Personalised advice from Hygienist		0.017	0.010	0.022	0.021	-0.028	0.058
12-m PI from Dentist		-0.007	0.062	-0.021	-0.009	0.036	0.030
12-m PI from Hygienist		0.013	-0.032	0.027	-0.025	-0.082 *	0.130 **
6-m PI from Dentist		0.020	-0.001	0.103 **	0.053	0.012	0.055
6-m PI from Hygienist		0.001	-0.004	0.071 *	0.069 *	0.033	0.025
Bleeding gums – Hardly ever		-0.022	0.045	0.069	0.098 **	-0.018	0.033
Bleeding gums – Occasionally		-0.046	-0.075 *	0.004	0.076 **	-0.018	0.110
Bleeding gums – Fairly often		-0.036	0.094	-0.080	-0.059	0.057	-0.026
Bleeding gums – Very often		0.057	-0.024	-0.075	-0.204 **	-0.083	-0.160
Teeth look and feel – v. unclean		0.187	0.028	-0.334 ***	0.117	-0.004	-0.213
Teeth look and feel - unclean		-0.077	0.124 *	-0.216 ***	-0.067	-0.003	-0.106

Teeth look and feel mod. clean		-0.111 ***	-0.047	0.160 ***	-0.047	-0.004	0.104
Teeth look and feel - clean		0.070	-0.102 *	0.177 ***	-0.014	-0.020	0.140 *
Annual cost		0.002 ***	0.000	-0.002 ***	0.001 ***	-0.001 **	0.002 ***
ASC *(Mean)*		0.307 ***	-0.024	-0.064	0.081	-0.134 *	0.320 ***
**Likelihood Ratio Test**							
Likelihood Ratio	2176	2068	2177	2163	2132	2148	2025
Chi^2 (df=16)		86.44	16.82	93.27	42.23	20.02	81.19
P		0.0000	0.3976	0.0000	0.0004	0.2195	0.0000

[a] Experience of hygienist: (1= ever visited a hygienist; -1 = never visited); [b] Region: (1= Scotland; -1=Rest of UK); [c] Sex: (1= Female; -1= Male); [d] Income: (1= Moderate / high income reported, ≥ £20,800 per year; -1 = Low income <£20,800 per year); [e] Smoker (1 = Current or previous smoker; -1 = Never smoked); [f] Experience of Scale and polish: (1= Ever had a Scale and polish; -1 = Never had a Scale and polish).
